# Comparison of targeted percutaneous vertebroplasty and traditional percutaneous vertebroplasty for the treatment of osteoporotic vertebral compression fractures in the elderly

**DOI:** 10.1186/s13018-020-01875-4

**Published:** 2020-08-26

**Authors:** Lingli Yuan, Jianzhong Bai, Chunhui Geng, Guansheng Han, Wendi Xu, Zhongchuan Zhang, Hong Luo, Xunbing Zhu

**Affiliations:** Department of Orthopedics, The Second Affiliated Hospital of Bengbu Medical College, Bengbu, 233000 China

## Abstract

**Objective:**

To investigate the clinical effect of precise puncture and low-dose bone cement in percutaneous vertebroplasty (PVP).

**Methods:**

Sixty patients with osteoporotic vertebral compression fracture (OVCFs) who were treated with PVP in our hospital from July 2018 to June 2019. These included patients were divided into group A (*N* = 30) and group B (*N* = 30). Group A has punctured to the fracture area accurately and injected with a small dose of bone cement, the group B was injected with a conventional dose of bone cement. The operation time, the amount of bone cement injection, the number of X-rays, the VAS scores, the leakage rate of bone cement, and the incidence of adjacent vertebral fractures were compared between the two groups.

**Result:**

The operation time, fluoroscopic times, and bone cement volume in group A are less than that in group B (*P* < 0.05). Patients in group A had a lower incidence of cement leakage and adjacent vertebral fracture than that in patients in group B. There was no significant difference in postoperative pain relief between the two groups.

**Conclusions:**

Precise puncture and injection of small doses of bone cement can reduce the number of X-ray fluoroscopy, operation time, amount of bone cement injection, reduce the rate of bone cement leakage and the incidence of adjacent vertebral fractures, which is a safe and effective surgical approach for the treatment for the aged with OVCFs.

## Introduction

With the aging of society, the treatment of osteoporosis has become a significant issue [1]. One of the most common complications of osteoporosis is osteoporotic vertebral compression fractures (OVCFs), especially in the place of thoracolumbar joint. Therefore, OVCFs always accompanied by spinal deformity, restriction of abdominal and thoracic contents, impaired mobility, and persistent intractable pain. Nowadays, it is estimated that almost 200 million older people are suffering from the disease [2]. Although only one-third of these fractures become symptomatic OVCFs, which is often related to decreased quality of life, increased disability, and mortality in the elderly [3]. Currently, there are many conservative methods for the treatment of symptomatic OVCFs, including bed rest, analgesics, bracing, antiresorptive medications, and a combination of these treatments initially. As the majority of patients are old people, prolonged bed rest often leads to some terrible complications, such as further loss of bone mass, deep venous thrombosis, and pneumonia. Anti-inflammatory drugs and analgesics often bring some severe side effects, which are difficult to tolerate for old patients. Besides, traditional surgical fixation was often invalid due to the poor quality of osteoporotic bone.

PVP was first performed for the treatment of a hemangioma in 1984 by Galibert and Deramond [4]. This invasive procedure involves augmentation of the OVCFs using a cannula injection of cement into the vertebral body directly under the perspective of imaging. However, bone cement leakage is one of the most common complications of PVP [5, 6]. Although the injection of a large amount of bone cement restores the height of the vertebral body, it increases the possibility of bone cement leakage, and postoperative pain relief was not significantly associated with bone cement injection [7]. PVP assisted by preoperative computed tomography and intraoperative X-ray, we can accurately penetrate the guide needle into the vertebral fracture area, and we injected a small-dose of bone cement in the fracture area. We called this new approach as the targeted PVP. The purpose of this study is to investigate the safety and effectiveness of this new surgical approach.

## Materials and methods

Sixty patients with OVCFs hospitalized in the Second Affiliated Hospital of Bengbu Medical College were enrolled in this prospective study from July 2018 to June 2019, informed consent has been signed for all included patients. Seventy patients were assigned to the targeted PVP group (group A) and traditional PVP group (group B), and 30 patients in group A, 30 patients in group B.

### Selection criteria

Inclusion criteria: (1) elderly patients with osteoporosis more than 60 years old, (2) single-segment thoracolumbar fractures, MRI showed fresh vertebral compression fractures, intact posterior wall of the fracture, and no symptoms of nerve injury; (3) all included patients had significant low back pain, visual analog scale (VAS) is more than 5 points.

Exclusion criteria: (1) multi-segment thoracolumbar vertebral compression fracture, single vertebral compression is more than two-thirds of the original vertebral body height, (2) pathological fractures, including tumors, hemangioma, etc., (3) patients with infection in surgical area; (4) patients with spinal stenosis, coagulopathy, old fractures, incomplete vertebral posterior wall, or nerve injury symptoms.

### Surgical technique

#### Group A

The patient is in the prone position, and the soft pillows on both sides of the front chest and the underarm cushion make the abdomen vacant to reduce the compression of the abdomen; the C-arm X-ray machine was used to observe the target vertebral body, and the upper and lower endplates are in a line. After fluoroscopy, the body surface is positioned and marked. Conventional disinfection drape, anesthesia with local infiltration of 1% lidocaine, deep into the periosteum around the pedicle. When anesthesia is sufficient, the waist is over retracted to restore the compressed vertebral body.

**Fig. 1:**
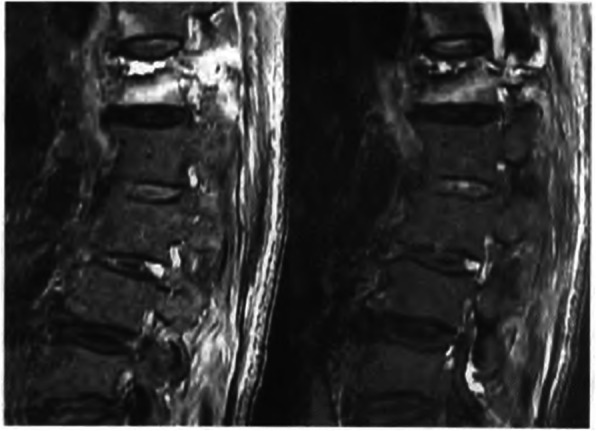
Preoperative MRI film of L_1_ vertebral compression fracture

**Fig. 2:**
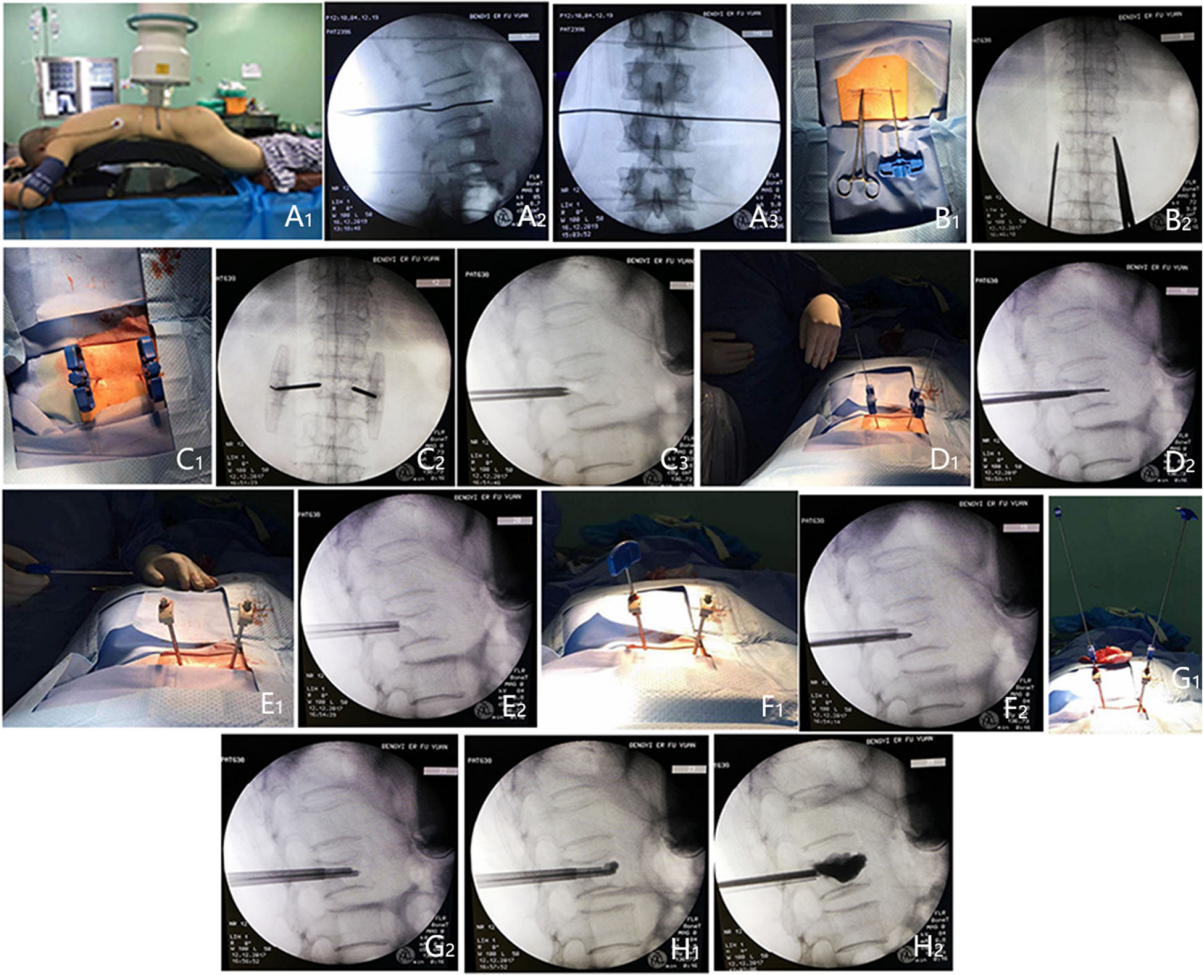
The specific steps of the operation (A_1-3_: positioning vertebral body; B_1,2_: positioning the pedicle; C_1-3_: puncture the fractured vertebral body; D_1,2_: implant the guide pin; E_1,2_: replace the outer tube; F_1,2_: tapping; G_1,2_: implanted cement thruster; H_1,2_: inject bone cement)

**Fig. 3:**
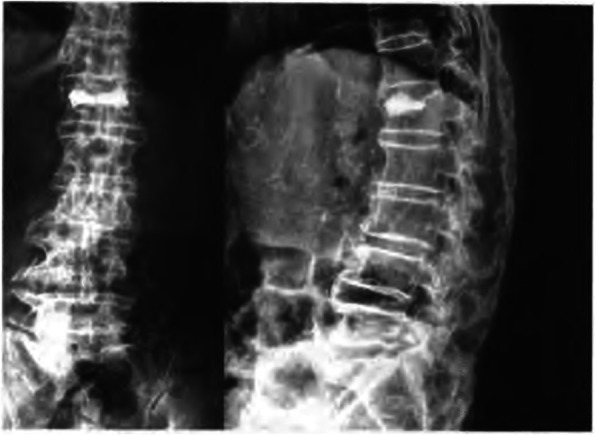
X-ray film after PVP shows a small amount of bone cement in the OVCF

The skin and subcutaneous soft tissue were cut with a scalpel at the marked location, the length of incision is about 0.5 cm. Under fluoroscopy, the core puncture needle was placed into the pedicle (the left pedicle was 10 points outside, and the right pedicle was 2 points outside). Adjust the puncture needle tilt or tail tilt angle according to the fracture line area. Under the lateral fluoroscopy, it is confirmed that the puncture needle is located in the pedicle, and continues to puncture the vertebral body to the posterior one in three of the vertebral body. At this time, the fluoroscopy needle tip is located slightly inside the inner edge of the pedicle shadow. This process requires precise penetration into the fracture line area, then exits the puncture needle, and the hollow drill takes a biopsy. An injection volume of bone cement was 2 to 3 ml per segment in thoracic vertebra and 3 to 4 ml in lumbar, respectively. Stitching incision and covered with a sterile applicator, postoperative prone position for 15 min after surgery to better coagulates bone cement. Preoperative MRI film of L_1_ OVCF is shown in Fig. 1, the specific steps of the operation are shown in Fig. 2, X-ray film after PVP shows a small amount of bone cement in the OVCF is shown in Fig. 3.

#### Group B

The fracture reduction and puncture method are the same as those in the observation group. The puncture needle does not have to be penetrated into the fracture line area. The amount of bone cement injected is 4 to 10 ml.

### Clinical outcomes

There was no significant difference in the general condition of the two groups of patients (Table 1). All patients were followed at least 6 months. Compared with group B, group A used shorter operation time, less bone cement, and fewer fluoroscopy times (*P* < 0.05, Table 2). There was no significant difference in VAS scores between the two groups at 2 days, 3 months, and 6 months after surgery (Table 3). In group A, 2 cases (6.7%) had cement leakage, 2 cases (6.7%) had adjacent vertebral body fractures. While in the control group, 7 cases (23.3%) had cement leakage, and 5 cases (16.7%) adjacent vertebral fractures (Table 4). The cement leakage rate of group A and the incidence of adjacent vertebral fractures were lower than group B.

**Table 1:** General characteristics of the patient

	Group A	Group B	*P* value
Year	75.5 ± 5.5	74.5 ± 4.5	> 0.05
Gender (M/F)	8/22	7/23	> 0.05
Weight (kg)	65.5 ± 4.3	66.5 ± 3.5	> 0.05
Height (cm)	165.4 ± 4.2	167.5 ± 3.8	> 0.05
OVCF level			
T11	1	2	> 0.05
T12	9	10	
L1	10	8	
L2	5	4	
L3	3	2	
L4	2	2	
L5	1	2

**Table 2:** Statistical comparison between group A and group B

	Fluoroscopic times	Bone cement volume (ml)	Operation time (mins)
Group A	20.3 ± 1.7	3.5 ± 0.5	25.8 ± 4.3
Group B	30.5 ± 2.4	8.5 ± 0.8	44.5 ± 5.8
*P* value	< 0.05	< 0.05	< 0.05

**Table 3:** Comparison of postoperative VAS between two groups

	Postoperative VAS (2 days)	Postoperative VAS (3 months)	Postoperative VAS (6 months)
Group A	5.3 ± 0.4	3.6 ± 0.5	0.9 ± 0.6
Group B	5.7 ± 0.8	4.1 ± 0.8	0.8 ± 0.7
*P* value	> 0.05	> 0.05	> 0.05

**Table 4:** Incidence of postoperative complications in two groups

	Group A	Group B
Cement leakage	2 (6.7%)	7 (23.3%)
Adjacent vertebral fracture	2 (6.7%)	5 (16.7%)
Complication rate (%)	13.3%	40.0%

## Discussion

The main cause of clinical symptoms after OVCFs is fractured trabecular micro-motion [8]. After the vertebral body fracture, the center of gravity will move forward. The load on the anterior and middle column of the vertebral body will increase, and the stress concentration will easily cause the adjacent vertebral body fracture and increases the risk of disk herniation. As the degeneration progresses, the posterior stretch stress of the spine increases, and the posterior margin of the vertebral body and the corresponding peripheral ligament hypertrophy can cause the corresponding segmental spinal canal stenosis. The clinical manifestations of the patient were progressive height loss of the vertebral body, aggravation of kyphosis, and intractable low back pain. As the kyphosis deformity, progressively, the degree of spinal activity decreases, the body balance and healthy posture are destroyed, resulting in a decrease in the volume of the thoracic cavity, a reduction of vital capacity, and some patients even have gastrointestinal symptoms. PVP is widely used in clinical practice because of its simple operation, small trauma, better curative effect, and rapid recovery [9]. It is currently the standard surgical method for the treatment of OVCFs. The mechanism of pain relief in PVP: (1) bone cement cures rapidly, eliminates fretting between fractures, increases stability between vertebral fractures [10]; (2) restores vertebral height, corrects kyphosis, and improves biomechanical properties; (3) the exotherm and toxicity of the cement when it is cured destroys the sensory nerve endings in the vertebral body [11].

Lu et al. considered that the distribution of bone cement is the main factor affecting clinical efficacy [12]. After solidification of the bone cement, it mainly acts as a stable trabecular bone trabeculae, supports the vertebral body, disperses the internal pressure of the vertebral body, and improves the mechanical distribution of the vertebral body. Accurate puncture into the vertebral fracture area, small-dose bone cement fixed fracture block, and achieve the purpose of fracture block solidification, clinical symptoms can be improved. Our study showed that the precise puncture of low-dose bone cement PVP in the treatment of osteoporotic vertebral compression fractures, the postoperative pain relief was similar to the B group.

In vitro mechanical tests by Belkoff et al. have shown that the infusion 2 ml of bone cement restores the strength of the vertebral body, while restoring the stiffness of the vertebral body requires at least 4 ml [13]. Cotten et al. investigated the relationship between the filling rate of bone cement in the vertebral body and the analgesic effect and found that the postoperative pain was significantly improved in patients with poor bone cement filling effect [14]. Therefore, there may be no significant correlation between the degree of pain relief and the filling rate of bone cement in the vertebral body. The pursuit of vertebral body maximum bone cement injection, recovery of vertebral height, and bone cement filling rate will increase the risk of bone cement leakage.

Bone cement leakage is a common and severe complication of PVP. The bone cement will leak into the spinal canal and burn the nerve root and spinal cord. After the bone cement leaks, pulmonary embolism and other important organ embolisms will occur. The previous study found that injecting 7 ml of bone cement into the vertebral body, the pressure in the vertebral body can be increased by sixfolds, and an increase of pressure in the vertebral body will directly lead to leakage of bone cement [15]. In our study, 7 cases of cement leakage in group B. In group A, two cases of cement leakage occurred. However, no venous leakage or spinal canal leakage happened in the two groups after surgery. According to literature reports, elderly patients undergoing PVP surgery for the first time have a risk of fracture of adjacent segments of the vertebral body of about 19.2%, and about 50% to 67% within a year [16], mainly due to the injection of bone cement in the vertebral body with higher hardness. The mechanical load is transferred to adjacent vertebrae, which increases the incidence of fractures in adjacent vertebrae. Sun et al. consider that the leakage of bone cement through the upper and lower endplates of the fractured vertebra to the intervertebral disk leads to increased stress in the adjacent vertebral body, which eventually causes the adjacent vertebral body to fracture [17]. The study of Lu et al. demonstrated that changes in disk pressure caused by leakage of bone cement disks may cause deflection of adjacent vertebral endplates, which may result in fractures of adjacent vertebral bodies [18]. Berlemann and other studies have shown that the hardness of the vertebral body changes after the injection of bone cement, which affects the mechanical conduction, weakens the endplate cushioning capacity, and increases the stress on the endplates and intervertebral disks of adjacent vertebrae. This is the leading cause of vertebral fractures in adjacent segments after vertebroplasty [19]. In this study, one adjacent vertebral body fracture was found in group A after surgery, and three adjacent vertebral body fractures were found in group B.

Huang et al. considered that adjacent vertebral fractures after PVP or PKP are a natural progression of osteoporosis [20], the severity of osteoporosis is a risk factor for secondary vertebral fractures after PVP [21]. Bone cement material and filling volume are also a risk factor affecting the occurrence of fractures in adjacent vertebrae. It was found that the rigidity of the vertebral body injected with silicone resin bone cement is closer to the rigidity of the human body. Silicone resin is a feasible option for the treatment of osteoporotic fractures, which has biomechanical potential to reduce the risk of secondary adjacent vertebral fractures [22]. Gilula et al. used cortoss cement and PMMA (polymethyl methacrylate) bone cement for surgery, and found that the incidence of secondary adjacent vertebral fractures in the cortoss cement group was lower than that in the PMMA bone cement group [23]. The optimal amount of bone cement is still controversial. The study of Liebschner et al. suggested that the injection of about 2 ml of bone cement can restore the strength of the diseased vertebral body to the level before injury. The amount of bone cement reaches 30% of the volume of the vertebral body, the rigidity of the vertebral body increases to 1.5 times the original level [24]. This study is similar to our findings, and low-dose bone cement also has a better therapeutic effect. Besides, the location, number, and age of the first fracture are also risk factors for adjacent vertebral fractures [25–27].

Reducing the amount of bone cement injected can reduce the risk of leakage [28]. Before the introduction of precise injection of small doses of bone cement, other methods can also reduce the leakage of bone cement. Increasing the viscosity of bone cement can better control the injection pressure and reduce the incidence of leakage [29, 30]. However, for the traditional bone cement injection group, we used a stepwise injection of bone cement. First, slowly inject about 1 mL of bone cement into the anterior middle one-third of the vertebral body, and observe the distribution of bone cement under the fluoroscopy. When the bone cement is close to the solidified state, the prepared bone cement is slowly injected into the vertebral body under fluoroscopy, and the injection is stopped when it approaches the posterior wall of the vertebral body.

Clinical application experience of accurate puncture to the fracture area and injection of low-dose bone cement PVP: (1) preoperative patients need to check MRI to determine whether the diagnosis is a fresh vertebral compression fracture; (2) CT three-dimensional imaging is used to determine whether there are cracks in the upper and lower endplates of the vertebral body. Whether the posterior wall of the vertebral body is ruptured, the possibility of leakage of bone cement, the condition of the pedicle, and the angle and position of the puncture are judged in advance, and the position of the fracture line is further determined to facilitate accurate puncture into the area of the fracture line; (3) under the premise of ensuring that the puncture needle penetrates into the fracture line, the thoracic spine is injected with 2-3 ml of bone cement and the lumbar spine is injected with 3-4 ml; (4) the patient was bedridden for 6-8 h after operation.

## Conclusions

Precise puncture and injection of small doses of bone cement can reduce number of X-ray fluoroscopy, operation time, amount of bone cement injection; reduce the rate of bone cement leakage and the incidence of adjacent vertebral fractures, which is a safe and effective surgical approach for the treatment for the aged with OVCFs.

## Data Availability

All data are fully available without restriction.

## Abbreviations

PVP: Percutaneous vertebroplasty
OVCF: Osteoporotic vertebral compression fracture
PMMA: Polymethyl methacrylate
VAS: Visual analog scale
